# Clinical and health policy challenges in responding to the COVID-19 pandemic

**DOI:** 10.1136/postgradmedj-2020-138027

**Published:** 2020-05-13

**Authors:** Donald Singer

**Affiliations:** Fellowship of Postgraduate Medicine, London, UK

**Keywords:** health policy, epidemiology, infection control

Severe acute respiratory syndrome coronavirus 2 (SARS-CoV-2), the cause of the coronavirus disease 2019 (COVID-19) pandemic, is the worst challenge for a century for international health and financial systems. It was declared a global pandemic on 11 March 2020, 6 weeks after it had first been reported from China as a new respiratory virus.^1^ By then, 118 000 cases had been reported from 114 countries and 4291 people reported to have lost their lives.^1^ Only 7 weeks later, as of 5 May, **3 544 222** cases of COVID-19, including **250 977** deaths, have been reported from 187 countries and regions, and maritime quarantine.^2^

While severity and mortality have been highest in people with underlying morbidities,^3^ no age group is immune from COVID-19 nor are the rich and famous. Reasons are unclear for more severe disease in males and, at least in the UK and USA, in ethnic minority groups. Members of many governments have been affected, including the British Prime Minister Boris Johnson, now discharged from hospital after a spell in intensive care. Reported mortality varies widely between countries with apparently similar economic development.^4^ Influences on reported case fatality ratios—the number of deaths divided by the number of reported cases—include the number tested, who is tested, test accuracy, demographics for age and comorbidity, and capacity and standards of healthcare staff and facilities. More reliable data, reported mortality, on 5 May ranged in the worst affected countries in Europe, for example, from 80 in Germany to 423 in the UK, 481 in Italy and 684 in Belgium per million in the general population^2^ and in North America 102 per million in Canada but 204 per million in the USA.^2^ These figures may reflect considerable underestimates of actual mortality, as deaths from COVID-19 among care home residents and deaths at home are typically not included.

National and international responses to COVID-19 are proving exacting tests of how effectively science and politics can work together to protect the public health—and wealth—of nations. In our globally connected world, an obvious expectation is that citizens are protected from avoidable risk from communicable diseases. Humanitarian expectations extend to ensuring that less developed countries are also able to cope with epidemics. Public health approaches have included a portfolio of measures including border controls, restrictions on national and international travel, isolating the public at home, except for essential workers, quarantining contacts of affected patients, complimented by diagnostic testing, health screening, contact tracing and use of surveillance apps.^5^ There has also been dramatic scaling up of provision of intensive care facilities through, for example, use of conference centres as temporary hospitals in the UK, to new hospital building in China and field hospitals, for example, in Central Park in New York City. There has also been major recruitment internationally of medical students and retired health professionals to help contact, trace and manage patients with active COVID-19 infection.

However, many diagnostic tests and digital health solutions are unreliable and are in use without proper evaluation.^6^ There are also concerns about surveillance apps about the trade-off between health versus privacy.^6^

There are serious gaps in response to the disease even in highly developed economies and healthcare systems. In the UK for example, it appeared to take modelling data from Ferguson’s group^7^ to persuade the government and its advisors to move rapidly from a ‘herd immunity’ stance to a national lockdown strategy. The delay in China’s reporting early cases did not help.^8^ Nor has the now revealed under-reporting to international public health authorities of mortality in China—at least 50% higher than initially reported.^9^ ‘Fake news’ has also complicated public responses to COVID-19 in many countries. This ranges from considering the virus the result of bioterrorism, to a disease caused by 5G wireless masts. Fake medicines are also a concern with (typically internet) vendors exploiting fears and concerns by falsely claiming that their products can treat or prevent COVID-19.^10^

To date, South Korea, which has a stringent detect, test, isolate, treat and contact trace policy, has reportedly had the greatest success in containing COVID-19. In the 14 days to 5 May, South Korea reported 2.4 new cases of COVID-19/million population and since the start of the pandemic 5.0 COVID-19 attributable deaths/million population, compared for example with the USA which, in the same two week period, reported 1203 new cases of COVID-19/million population and 204 COVID-19 attributable deaths/million population since the start of the pandemic.^2^

The European Union (EU) is showing its capacity to co-ordinate responses at several key levels. The EU’s Centre for Disease Prevention and Control is an important resource for information about the virus.^2^ The EU is also co-ordinating member states in consortia aimed at commissioning essential medical supplies. The EU regulator, the European Medicines Agency, is working with other regulators, including the US Food and Drug Administration, to support research and development of new treatments, from vaccines for disease prevention to new or repurposed medicines for use during active SARS–COVID-19 disease. Among over 100 candidate treatments for COVID-19, the following currently authorised medicines are already undergoing clinical trials of their safety and effectiveness: the anti-HIV medicines lopinavir/ritonavir, chloroquine and hydroxychloroquine (authorised as anti-malarials and as anti-inflammatory treatments for autoimmune diseases, eg, rheumatoid arthritis), the investigational anti-viral agent remdesivir, and interferons and immune-modulating monoclonal antibodies.^10^ Several vaccines are already in phase I clinical trials in healthy volunteers.^10^ However, based on previous experience of vaccine development, even the new GSK–Sanofi vaccine partnership^11^ estimates that 12–18 months may be needed to provide adequate supplies of effective vaccines for the EU region alone.

Personal protective equipment (PPE: masks, gowns, gloves and eye protection) for health professionals should be of high quality, be personalised for fit and be changed between patients. Among highly developed countries, the UK appears to be particularly unsuccessful in providing international standard PPE in sufficient quantities for acute healthcare staff and for the social care sector. Early approaches by UK manufacturers to provide supplies appear to have been largely ignored by the UK government in favour of international sources which, many weeks into the pandemic, have not as yet proved to be able to meet essential UK demand.^12^ The UK has also been very late in engaging with EU-led commissioning consortia to secure further PPE supplies and reportedly too late to join EU-led approaches to secure ventilators.^12^ There are continuing widespread reports in the UK of health professional staff not being provided with adequate PPE. If these reports are correct, the consequences are unacceptable—avoidable deaths from COVID-19 in health professionals and their unaffected patients and social contacts.

Use of face masks by the public is customary within East Asia. Concerns elsewhere about their use by the public include mask quality, over-confidence leading to less attention to social distancing, and with masks being in scarce supply, reduced availability for health professionals. Equipoise in other developed countries is however moving towards the precautionary principle.^13^ Face masks, for example, appear more likely to reduce risk of viral transmission, limiting particulate spread during speech, coughing and sneezing.^13^

The UK administration is not atypical in having prioritised economics over public health in rejecting recommendations of scientists within pandemic preparedness initiatives. In 2005, the then US President George W Bush launched an unsuccessful call at the US National Institutes of Health for a three-part approach, involving raising public awareness about epidemics and action needed, stockpiling PPE and other supplies, and acquiring rapid systems to develop treatments against major threats from communicable disease.^14^

An expected side effect of economic downturn because of COVID-19 has been a remarkable decrease in atmospheric pollution,^15^ a well-recognised contributor to severity of many clinical disorders, from heart and lung disease to cancers. Outcomes from COVID-19 appear worse in people historically exposed to atmospheric particulate pollutants and the inflammatory gas nitrogen dioxide.^15^ This should contribute to evidence to influence political support for continued reduction in harmful emissions into the atmosphere to reduce the severity of any future recurrent waves of COVID-19.

International co-ordination is inconvenient for countries where business and other interests are pushing for early relaxation of public health controls. The USA is doing its best to undermine the WHO as a forum to plan for resolving the COVID-19 pandemic and for better preparedness for future pandemic infections.^16^ Tan and his colleagues from Toronto have put the case for a new dedicated international forum for pandemic preparedness.^17^

There are many questions to be answered by virologists, epidemiologists, geneticists, pharmacologists and other scientists. How did the virus become a human pathogen? The zoonotic transmission route for the virus is still unclear, let alone how to disrupt it. Identifying this is a priority, given the zoonotic origins of so many historical epidemics of communicable diseases. Scientific research is also needed into how the virus reproduces in the body, how it interacts with the immune system and risk factors that contribute to disease of severity.

Experience of COVID-19 across the world indicates that pandemic preparedness in most countries appears at best to have been a paper exercise. Stockpiling essential medical supplies and having reserve health service capacity are undoubtedly costly. But so are the consequences for facing a pandemic unprepared. Developing vaccines and other treatments against an as yet unknown pathogen takes time. However, a much lower cost action could and should have been prepared in advance: achieving health literacy about pandemics in the population to support having the public ‘on side’ with necessary societal restrictions. Lack of this was reflected for example by Ferguson’s group including a high public non-adherence factor in their models for the UK,^7^ and in the USA there have been armed demonstrators in the streets in Michigan protesting against restrictions on their activities.

At this stage, it is too early to be clear about the longer-term severity and persistence of COVID-19 and therefore how long current public health controls should remain in place. However, early relaxation of social controls in some regions appears to be leading to a significant rise in incidence of the disease, for example, in Singapore and Japan, with reports also of significant COVID-19 resurgence in China.^2^

It remains to be seen how well the world’s financial systems and businesses will survive the pandemic and how long it will take to emerge from the current major economic downturn. In prospect are sustained increases in remote ways of working, within business sectors generally, as well as within health services. This journal will continue to report on the implications of the COVID-19 pandemic and welcomes manuscripts on how best to be prepared for future epidemics and pandemics.
